# What Are Punishment and Reputation for?

**DOI:** 10.1371/journal.pone.0045662

**Published:** 2012-09-26

**Authors:** Max M. Krasnow, Leda Cosmides, Eric J. Pedersen, John Tooby

**Affiliations:** 1 Center for Evolutionary Psychology, University of California Santa Barbara, Santa Barbara, California, United States of America; 2 Department of Psychological and Brain Sciences, University of California Santa Barbara, Santa Barbara, California, United States of America; 3 Department of Anthropology, University of California Santa Barbara, Santa Barbara, California, United States of America; Ecole Normale Supérieure, France

## Abstract

Why did punishment and the use of reputation evolve in humans? According to one family of theories, they evolved to support the maintenance of cooperative group norms; according to another, they evolved to enhance personal gains from cooperation. Current behavioral data are consistent with both hypotheses (and both selection pressures could have shaped human cooperative psychology). However, these hypotheses lead to sharply divergent behavioral predictions in circumstances that have not yet been tested. Here we report results testing these rival predictions. In every test where social exchange theory and group norm maintenance theory made different predictions, subject behavior violated the predictions of group norm maintenance theory and matched those of social exchange theory. Subjects do not direct punishment toward those with reputations for norm violation per se; instead, they use reputation self-beneficially, as a cue to lower the risk that they personally will experience losses from defection. More tellingly, subjects direct their cooperative efforts preferentially towards defectors they have punished and away from those they haven’t punished; they avoid expending punitive effort on reforming defectors who only pose a risk to others. These results are not consistent with the hypothesis that the psychology of punishment evolved to uphold group norms. The circumstances in which punishment is deployed and withheld–its circuit logic–support the hypothesis that it is generated by psychological mechanisms that evolved to benefit the punisher, by allowing him to bargain for better treatment.

## Introduction

Evolutionary biologists and economists have long recognized that, under some conditions, cooperative strategies yield large fitness payoffs (e.g., gains in trade, risk pooling) [1]–[3]. Indeed, over the last two decades, there has been a growing consensus among biologists, psychologists, economists, and cognitive neuroscientists that humans have evolved decision-making specializations designed to capture these cooperative payoffs [4]–[6]. This hypothesis is now supported by an increasingly diverse body of experimental [5], [7]–[11] and neuroscientific [12]–[15] evidence.

Yet surprisingly large disagreements persist about what actual strategies these cooperative specializations were designed by natural selection to execute. The primary consensus is a negative one: Many findings in experimental economics have falsified the hypothesis that human cooperative behavior is simply the product of standard game-theory-derived rationality (which assumes, e.g., that agents will act so as to maximize individual payoffs given a defined game structure) [16], [17]. Here we report experiments that critically test two major surviving theories of human cooperation against each other, by testing conflicting hypotheses about the cues the cognitive architecture uses and the behavioral outputs that it produces.

Although there are a number of evolutionarily based theories about how and why humans cooperate, including kin selection [18], [19], indirect reciprocity [20], and externality-based affiliation [21], here we focus on evaluating two leading but fundamentally different families of theory. One proposes that cooperative decision-making adaptations evolved primarily for small-scale–often dyadic–cooperation and that these adaptations were maintained by fitness benefits accruing directly to the interactants [2], [4], [5], [22]. The other proposes that, in cooperative interactions, the use of reputational information, punishment, and the extension or withholding of trust function to maintain group-beneficial cooperative norms within large-scale groups [23], [24]. We call the first Social Exchange theory, and the second Group Norm Maintenance theory.

For the purposes of this paper, we do not consider or evaluate ongoing debates concerning the mathematical foundations, game dynamics, or theoretical coherence of competing models of cooperation [25], [26]. Our goal is instead to test their predictive power [27]. To do this, we need to assume–for purposes of empirical assessment–that both theories are viable, and then identify the circumstances under which Social Exchange theory and Group Norm Maintenance theory make different empirical predictions. These arise from differences in how each theory characterizes (i) the functional outputs that their respective cooperative strategies are designed to produce (e.g., promoting group-beneficial norms vs. initiating and maintaining individually profitable personal relationships), and (ii) the specific decision-making designs and methods by which their payoffs are produced (e.g., punishing violators of group norms vs. punishing exploitive or underperforming partners).

For social exchange, the overarching adaptive problem is the cultivation and maintenance of individually profitable, enduring exchange (or reciprocation) relationships [21]. On this theory, punishment, the use of reputation, and the extension or withholding of trust result from decision-making adaptations designed to deliver net lifetime payoffs directly to the individual. These payoffs are produced by sequences of gains in trade with individual exchange partners–partners selected, with limited information, from a potential set whose members differ in how rewarding they would likely be to interact with. Adaptive subproblems that social exchange adaptations evolved to solve include (i) discriminating potential partners on the basis of how likely they are to cheat the decision-maker [28], (ii) navigating uncertainty over how many repeat encounters with a given individual there will be [29], and (iii) incentivizing better returns from partners by rewarding delivery of high payoffs and by punishing defections or low payoffs in an effort to bargain for better treatment in the future [30].

In contrast, the central adaptive problem for group norm maintenance theories is the cultivation and maintenance of group-wide cooperative norms–a very different functional product. According to these theories, punishment, the use of reputation, and the extension or withholding of trust result from decision-making adaptations designed to encourage cooperative gains globally for all the norm-abiding members of a group, while simultaneously making norm violation less profitable than norm upholding for all members of the group. While an individual may incur a personal cost by–for example–engaging in one-shot cooperation or 3^rd^ party punishment, Group Norm Maintenance theories argue that groups in which these behaviors are prevalent outcompete groups in which they are rare or absent, explaining (under certain conditions) the spread and maintenance of these behaviors [31]–[33].

The Social Exchange/Group Norm Maintenance debate has persisted for over a decade because empirical studies have produced data that are consistent with both theories. Consequently, differing interpretations are inconclusively advanced for the same data sets [34], [35]. For example, it is often observed that subjects are willing to donate to others, even when experimenters inform subjects that such donations will never be repaid, such as when decisions are made anonymously and interactions occur only once [16]. Although some have concluded that such behavior is evidence of pro-cooperative group-selected norms [31]–[33], this interpretation has been advanced without evidence that subjects in these experiments donate differentially towards members of their own group. Instead, evidence suggests that when subjects do prioritize their own group members, they do so not because they are in-group per se, but because they have an expectation of generalized reciprocity from in-group members [36]. Moreover, anonymity does affect the size of donations: people donate less to others when cues of true privacy are enhanced [37], [38].

Social Exchange theorists have offered an alternative interpretation of the same phenomena: they argue that one-shot donations are the predictable product of mechanisms designed to minimize the risk of alienating potentially profitable partners–ones who would view a lack of generosity as mistreatment of themselves or others [11]. At the time of an initial interaction, its status as a one-shot encounter is inherently unknown, uncertain, and mistake-prone; the interaction can only be discovered to be one-shot retroactively, after subsequent encounters fail to happen. On this view, the minor gains from exploiting others in single interactions do not compensate for the many rounds of cooperation lost from a repeat interaction misjudged to be one-shot. Small initial investments in noisy environments function as a means of acquiring relationships that, ancestrally, would have had the potential for long-term personal gain [21]. Indeed, modeling demonstrates that generosity like that observed in one-shot experimental interactions systematically coevolves with social exchange in social ecologies where there is uncertainty about how many times the interactants will encounter each other (as would have been true for our foraging ancestors) [29].

Because Social Exchange and Group Norm Maintenance theories make overlapping predictions about cooperative phenomena, such as nonzero cooperation in apparently one-shot interactions, demonstrating such phenomena cannot discriminate between them. Fortunately, the adaptive problems posed by social exchange and group norm maintenance are markedly different; thus, they predict architectures that implement different behavior-regulating solutions. Specifically, Social Exchange theory and Group Norm Maintenance theory make *opposing* predictions about the uses to which reputational information will be put, and about the conditions that should elicit trust, punishment, and the refusal of cooperative solicitations.

Note that neither Social Exchange nor Group Norm Maintenance theory are premised on the exclusion of the other theory; there is room in the brain for both Social Exchange and Group Norm Maintenance mechanisms and it is possible for both theories’ unique predictions to be simultaneously empirically expressed. However, neither theory can be validated by demonstrating phenomena that fall in their area of joint prediction. Support for either theory can come only from its unique predictions. Here we present two experiments designed so that Social Exchange and Group Norm Maintenance theories produce sharply divergent predictions–that is, where key behavioral measures will be different in direction, and not simply in magnitude, depending on which evolved strategies are implemented in the human cooperative architecture.

### Predicted Behavioral Outputs from a Social Exchange System

Under certain conditions, individuals who invest in cooperative relationships can achieve a selective advantage over individuals who do not [1], [2]. This advantage is fragile; it depends on adaptations that allow cooperative investments to be directed toward individuals who reciprocate cooperation and away from those who do not (cheaters). The system should direct cooperative effort toward a partner as a function of that person’s estimated payoffs to the decision-maker (rather than, e.g., to the group) over the life of the relationship. The suite of adaptations predicted by Social Exchange theory is designed to solve the adaptive problems inherent in dyadic or otherwise small-scale exchange relationships [4]. To begin with, in order to successfully reap the benefits of small-scale cooperation, an organism must forage for individually profitable cooperative relationships by (for example) taking risks to form or repair them. Indeed, models typically show that dyadic cooperation does better when it coevolves with a disposition to extend trust on first encounter [1]. However, because being defected on directly lowers the actor’s payoff to cooperation, the Social Exchange system should be designed to resist exploitation by detecting defection–as evidence shows it is [5]. Indeed, it is even better if the Social Exchange system can judge which candidates are likely to cooperate versus defect on the actor *before* actually interacting with them. All else equal, candidates with a disposition to cheat will have cheated others more often than candidates with a disposition to cooperate. While this record of past behavior with others (here called *3^rd^ party reputation*) does not guarantee that the candidate will treat the actor in the same way, it is a possible proxy cue for the true variable the Social Exchange system benefits by estimating: the probability the candidate will defect on the actor. Hence, the first prediction about the Social Exchange architecture is as follows: 3^rd^ party reputation should be used as a cue regulating trust when information about how the candidate has treated the actor (here called 1^st^ party reputation) is not available (Social Exchange prediction 1).

Using 3^rd^ party reputation to predict how a candidate will treat oneself poses a signal detection problem, however. It is costly for the actor to miss profitable dyadic opportunities with individuals just because those individuals did not cooperate equally well with everyone else (i.e., have been “norm violators” toward others). Information about behavior toward 3^rd^ parties is likely to supply some false alarms about a candidate’s behavior toward the self. What matters for a profitable social exchange strategy is how well a candidate will cooperate with the actor in particular, and reputation about 3^rd^ parties is only an imperfect cue to that likelihood.

The partner’s past behavior toward the actor is a better predictor of the partner’s future behavior toward the actor than is the partner’s past behavior toward 3^rd^ parties. Therefore, Social Exchange theory predicts that 1^st^ party information about the candidate’s value as a cooperative partner will be weighted more heavily than 3^rd^ party information in decisions to trust, and may be expected to override it (Social Exchange prediction 2). Recent research suggests that such noncompensatory use of cues (i.e., use of a less reliable cue only in the absence of a more reliable cue) may be a frequent feature of evolved decision systems [19], [39].

Most significantly, whereas Group Norm Maintenance predicts that punishment and the refusal of cooperative solicitations will be jointly deployed against norm violators (see below), Social Exchange theory predicts that one will instead be *more likely* to cooperate with defectors whom one has punished. According to Social Exchange theory, the cost imposed by defection can be countered in one of two ways: the actor can (i) withdraw from the relationship by refusing to cooperate in the future or (ii) continue the cooperative relationship but bargain for better treatment. In Social Exchange theory, punishment is a bargaining strategy; it is an investment in a potentially continuing cooperative relationship [40].

In order to ensure the profitability of ongoing cooperative relations, an organism must be sensitive to indicators of its current treatment and bargain to enforce a profitable standard of treatment. Social Exchange theory argues that in cooperative relationships, punishment (orchestrated by the evolved program of anger) is a way of rejecting unacceptable terms of division for joint gains in trade [30]: it communicates to the defector that, in order for the relationship to continue, the defector must improve her or his treatment of the punisher in future cooperative interactions. The actor’s return for such a costly investment in punishment is the potential reduction or elimination of the partner’s exploitive behavior in subsequent exchanges, allowing longer chains of personally beneficial gains in trade. On this view, the cost of (private) punishment can be recouped only if the cooperative relationship continues. Thus punishment should be deployed only to the extent the punisher anticipates or intends a continuation of the cooperative relationship. (Note: This argument is *not* that an individual’s costs are recouped by changes in others’ behavior every time he or she punishes a defection [41]; the attempt to bargain for better treatment may sometimes fail. As analogy, the observation that prey are sometimes caught while trying to escape predators is not evidence against the hypothesis that prey are designed to attempt escape.).

Actors who have decided to terminate their relationship with a defecting partner can realize no fitness advantage from punishing the defection. Those who have decided to withdraw from a relationship are expected to distrust and refuse cooperative solicitations by the defector. Because withdrawing cooperation anticipates (and initiates) the termination of one’s relationship with a partner, refusing cooperative solicitations should be negatively correlated with punishment.

For these reasons, Social Exchange theory predicts that actors are more likely to cooperate with a defector that they have punished than one that they have not. Thus there will be a negative relationship between punishment and the termination of cooperative investment (e.g., distrust, refusing cooperative offers, etc.; Social Exchange prediction 3).

### Predicted Behavioral Outputs from a Group Norm Maintenance System

Under certain circumstances, a homogeneous group of cooperative norm upholders can achieve selective advantage in competition with other groups [33]. This advantage is fragile, however; it requires cost-effective defenses against exploitation by norm violators. Group Norm Maintenance theories focus on solutions to this adaptive problem. As a suite of adaptations for maintaining cooperative norms, Group Norm Maintenance systems should pick out defectors (violators of cooperative norms) based on information diagnostic of their disposition to treat others in a norm-violating way. Because any single instance of norm violation could be the product of misunderstanding, mistake, or other circumstances that would mitigate against a dispositional attribution, multiple instances of norm violation should serve to strengthen the attribution of norm violator status, compared to a single instance [9]. This logic is generally relevant to all theories of cooperative psychology, Social Exchange theory included. However, Group Norm Maintenance diverges from other such theories by predicting that this pattern should hold for norm violations whether the victims are others, the self, or both; that is, a character assessment system designed for maintaining cooperative group norms should register cases in which an individual has defected on others as well as cases in which that individual defected on one’s self, because both are evidence that the individual has violated group cooperative norms. It would cease to be a *group* norm maintenance theory if the individual’s treatment of third parties did not count. Therefore, a fundamental prediction of Group Norm Maintenance theory is that the cue of being defected on by an individual should be integrated in some fashion with parallel information about that individual’s propensity for norm violations against others (Group Norm Maintenance prediction 1).

For clarity, we will use the terms ‘1^st^ party reputation’ and ‘3^rd^ party reputation’ to refer to these constructs: 1^st^ party reputation summarizes past cooperation-relevant acts done by the partner to the self; 3^rd^ party reputation summarizes past cooperation-relevant acts done by the partner to others. It is important to note that this is different from (and orthogonal to) the distinction between events individuals experience themselves and those that are reported to them second or third hand on the word of others. So when an individual is informed by third parties that she has been cheated by a specific transgressor, this information constitutes 1^st^ party reputation just as much as if the individual had directly observed the transgression for herself. This is because 1^st^ party reputation is about what the transgressor did to the self, regardless of the source of the information. Hence, it is important to understand that the experiments herein are designed so that both 1^st^ party and 3^rd^ party reputation are based on information supplied in precisely the same way: by the experimenter (rather than by direct experience). Any differences in how the two reputation types are treated by the mind cannot, therefore, be explained by invoking either direct experience or source credibility as factors, because these were held constant in these studies.

After identifying a group-norm violator, effective responses must be taken to make norm-violating lower paying than norm-upholding [33]. Group Norm Maintenance predicts that the degree to which an individual has been categorized as a norm violator should predict the degree to which they are targeted for norm-enforcing responses. On various versions of Group Norm Maintenance theory [24], [31]–[33], norm violators should be targeted for withholding of trust (Group Norm Maintenance prediction 2), application of punishment (Group Norm Maintenance prediction 3), and rejection of requests for cooperative treatment (Group Norm Maintenance prediction 4). Consequently, the degree to which these responses are made should be positively correlated (Group Norm Maintenance prediction 5), as they are all responses to the same provocation–the violation of a group norm. On these versions of Group Norm Maintenance theory, such actions could function to: (i) motivate the violator to uphold the norm in the future, (ii) dissuade others from imitating the norm violation, or (iii) exclude the violator from the fruits of group cooperation. Regardless of the proximate effect, however, the ultimate design of the Group Norm Maintenance system is to maintain within-group cooperative norms against the spread of otherwise higher paying exploitive strategies.

Testing between these contrasting sets of predictions requires an experimental design with several features not commonly found together. (i) Subjects must be exposed to reputational information about someone, and then have the chance to trust them in the context of a potentially cooperative interaction. (ii) Following cases of the partner defecting on the subject’s trust, the subject must have the chance to respond with punishment. (iii) Finally, the subject must have the chance to cooperate with their partner after making the decision to punish or not. The following two studies were designed to meet these criteria.

## Methods

### Ethics Statement

Informed consent was obtained in electronic written form and recorded in subjects’ data files. This, and all other recruitment and experimental protocols were approved by the University of California, Santa Barbara Human Subjects Committee.

### General Methods

The subjects in both studies were undergraduates from the University of California, Santa Barbara. Ninety-three subjects (65 females) participated in Study 1, and 119 subjects (71 females) participated in Study 2. Subjects were sampled from the general student population and recruited via printed or online advertisements. Subjects were brought into a computer lab and instructed that they would be interacting with others over the computer network. In reality, they interacted with sham partners, simulated by a computer script. This minor deception was necessary; it allowed us to tightly control individual subject experiences, ensuring that they had the opportunity to interact with all combinations of partner reputation and behavior required to test between Social Exchange and Group Norm Maintenance theories. (It may be helpful here to note that, while generally avoided by behavioral economists, deceptive methodologies are safely and effectively used in psychology and throughout the rest of the behavioral sciences because they can help to maximize experimental control and rigor [42]. At the end of the experiment, subjects were questioned for suspicion of deception; such suspicion was not found to predict any aspect of performance (see Appendix S3). Before leaving, subjects were debriefed and told they had been interacting with sham partners.).

The two studies were designed to test how 1^st^ and 3^rd^ party reputation information regulates decisions to trust, cooperate, and punish one’s partner. In both studies, reputation information about the partner was provided by the experimenter via the computer; trust, cooperation, and punishment were measured with a series of two-round trust games (TGs). Merely described to subjects as a social interaction in which real money was at stake, the games offered subjects the opportunity to earn a large return by investing a portion of their endowment with their partner. This investment was risky, however, as the benefit was contingent on the partner voluntarily returning an equal share of the gain. Should the partner withhold an equal share from the subject, the subject had the option to pay to punish the partner’s behavior. In the second round, the roles were reversed and the partner had the chance to invest with the subject. Before playing with each of four partners, subjects were given information about that partner’s reputation for cheating versus cooperating. In order to support the deception that subjects were interacting with a real partner, the same type of reputation information was first collected from the subject.

At the end of each study, subjects were probed to see if they suspected their partner was not a real person (see Appendix S2), and *any* mention of suspicion of the deception resulted in the subject being coded as suspicious. No subject who mentioned suspicion of the deception–and some were quite sure of it–indicated that it influenced their decisions. However, to test for any effects, suspicion of deception was entered as a subject level variable into each analysis and was not found to predict any aspect of subject performance (see Appendix S3). Further, each analysis was repeated omitting the suspicious subjects, yielding results inferentially identical to the full data set (see Appendix S3).

The overall structure of each study was as follows: (1) Information was collected about the subject’s propensity to cheat; subjects were not told that this information might be given to future partners. (2) Subjects were paired with a series of partners. For each partner, they first received information relevant to that partner’s propensity to cheat, and then played a two-round TG. (3) Subjects were then probed for suspicion of the deception and debriefed.

### Study 1

The purpose of this study was to test whether 3^rd^ party reputation regulates decisions to trust, cooperate, and punish one’s partner. Reputation information was derived from responses to a disposition-to-cheat questionnaire, consisting of hypothetical scenarios in which an individual indicates what they would do when given an opportunity to cheat without detection (see Appendix S1).

Subjects first answered the disposition-to-cheat questionnaire. The computer then informed each subject that he or she had been randomly selected to receive information about how their partners had answered the same questions (and that these partners would not have access to the subject’s answers). Before playing the TG with a given partner, that partner’s answers to four of the disposition-to-cheat scenarios were disclosed to the subject. Subjects encountered four partners who differed in their character profiles: in random order, subjects interacted with partners who chose the cheating option for zero, one, three, and four of the four scenarios.

**Figure 1:**
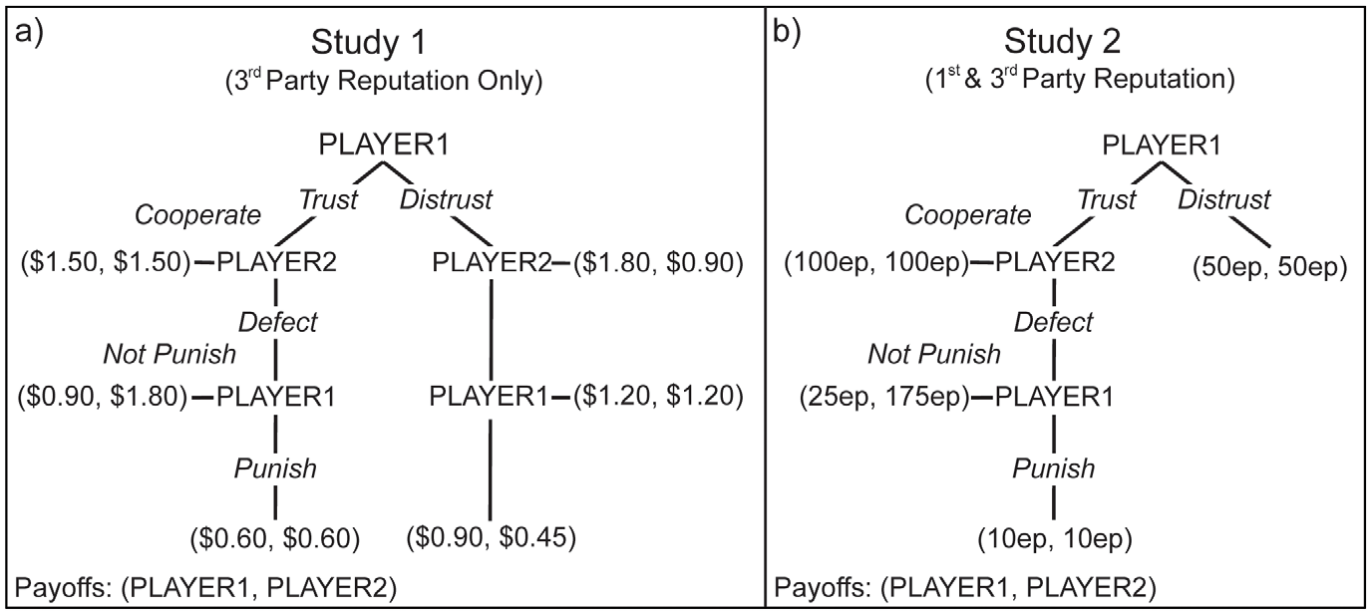
Two-Round Trust Game. Two-round trust game played by subjects after receiving partner’s reputation. In the first round, subjects were assigned to be Player 1 and could choose to move left (labeled “Trust” above) or right (labeled “Distrust” above). If trusted, the partner could reward the subject’s trust by choosing a symmetrically large payoff (labeled “Cooperate” above) or choose a self-favoring payoff (labeled “Defect” above) that yielded less for the subject than if they had initially moved right. If the partner defected, the subject then decided to either punish this decision, paying a small amount to impose a large cost on the partner (labeled “Punish” above), or choose the option with a higher payoff while allowing the partner to profit from his defection. In the second round, the roles were reversed and the partner made the initial decision. Note that decision labels were not displayed to subjects.

Subjects then played a two-round TG with each partner. The structure of the game is depicted in Figure 1a, and was adapted from Hoffman, et al. [9]. The computer informed subjects that they had been randomly assigned to the role of first mover in round 1, and second mover in round 2. In round 1, the subject’s first choice was to move left (trust) or right (distrust). Using backwards induction and assuming rational self-interest for both players, the subject’s expected outcome for trusting is $.90, compared to $1.20 for distrust. If their partner cooperates with them, then trusting could yield a higher payoff of $1.50, but this decision is risky: it gives the partner the opportunity to gain even more by defecting on the subject. If the subject trusts and the partner defects, the subject has the opportunity to either accept this selfish decision and earn $.90, or pay $.30 to punish their partner by $1.20, leaving each with $.60 for the first round. In the second round, the roles are reversed. The partner has the opportunity to trust the subject; if the partner trusts, then the subject has the opportunity to cooperate or defect. If the subject defects, the partner has the opportunity to punish. (Unbeknownst to subjects, sham partners used the strategy: In round 1 if the subject trusts, cooperate 50% of the time, and if the subject distrusts, follow rational self-interest; In round 2 trust 100% of the time, and if the subject defects then punish 33% of the time. These values were chosen to expose all of the theoretically relevant choice points to scrutiny, and otherwise conform to common human performance [9].).

### Study 2

The purpose of this study was to test whether 3^rd^ party reputation regulates decisions to trust, cooperate, and punish when information about 1^st^ party reputation is additionally available. Instead of responses to the disposition-to-cheat questionnaire, reputation information was based on how the partner had behaved in two standard Prisoner's Dilemma (PD) games. Subjects decided whether to cooperate or defect in a block of four standard Prisoner’s Dilemma (PD) games, one played with each of four partners (see Appendix S1), without feedback on their partners’ decisions. Before playing the TG with a given partner, the computer displayed that partner's decisions for two of the previous PDs: one with the subject and one with a 3^rd^ party. Thus the subject was given 1^st^ and 3^rd^ party reputational information about the partner, and the format and source of that information was held constant while only the target of the behavior varied. As in Study 1, Study 2 subjects also encountered four character profiles: a partner who defected on them but cooperated with another subject, one who cooperated with them but defected on another subject, one who cooperated with both, and one who defected on both. The order of partner-types was randomized across subjects.

Subjects then played a two-round TG with each partner. Subjects were informed at the outset that they would be playing for experimental points (ep), and that all their earnings from the games would be converted to dollars and paid to them at the rate of 300ep  =  $1. Subjects earned a $3 show-up payment in addition to their other earnings.

The structure of the TG was simplified from Study 1 by summarizing the right branch decisions into a single outcome (Figure 1b). For the first round, the subject was given 100ep; they could split them evenly (keeping 50ep and ending the round) or make a risky investment by passing the endowment to their partner, in which case it was multiplied to 200ep. On receiving the endowment, the sham partner could cooperate, returning 100ep to the subject (keeping 100ep), or defect, returning only 25ep (keeping 175ep). If the return was selfish, the subject could spend 15ep to punish the partner by 165ep, yielding a mere 10ep for each in that round. In the second round, the roles were reversed; the partner has the opportunity to trust the subject, the subject then has the opportunity to cooperate or defect, and if the subject defects, the partner has the opportunity to punish. (As in Study 1, sham partners used the strategy: In round 1 if the subject trusts, cooperate 50% of the time; In round 2 trust 100% of the time. To better approximate human performance from Study 1, the punishment rate was modified from 33% to 40%.).

### Data Analysis

Subjects’ game behavior was modeled (using Scientific Software International’s HLM software) as separate hierarchical logistic models predicting decisions to trust, punish, and cooperate. In these models, game-level predictor variables (such as partner’s reputation and game behavior) were nested under subject-level predictor variables (such as sex and the subject’s own responses to the character assessment instrument). Key results that discriminate between the predictions of Group Norm Maintenance and Social Exchange theories are listed below (see Appendix S3 for the full model estimations and coding scheme).

## Results and Discussion

The results of these studies are strikingly clear: In none of the critical tests–those where social exchange theory and group norm maintenance theory made divergent predictions–was Group Norm Maintenance theory supported. Group Norm Maintenance theory’s predictions were only supported in those few cases where Group Norm Maintenance and Social Exchange made the *same* predictions (and hence received the same support). In contrast, the pattern of results unambiguously supports Social Exchange theory’s predictions about the interlocking functions of trust, reputation, and punishment. The two studies not only replicate each other’s core findings, but they do so using very different forms of reputation–the partner’s own disclosure of his or her willingness to cheat in several real-world scenarios (Study 1) and the partner’s behavior in response to Prisoner’s Dilemmas (Study 2). These represent a wide spectrum of forms that reputation information may take, yet they elicited robust and remarkably similar results. Together, these studies answer the following questions:

### Are Decisions to Trust Regulated by 3^rd^ Party Reputation when Subjects have no Information about 1^st^ Party Reputation?

Yes. Study 1 tested this, as here the only reputation available was the target's professed willingness to cheat 3^rd^ parties. On the first move, subjects in round 1 could choose to either trust their partner (choosing the left branch of the decision tree) or protect themselves against defection (right branch), and subjects made this mistrusting choice 36% of the time. In this situation, the rate at which subjects chose the option that protected them from defection was directly proportional to the number of times the partner indicated willingness to cheat 3^rd^ parties, Odds Ratio (OR)  =  1.63 *t_363_* = 7.02, *p* = 10^−11^. Or put otherwise, partners with a reputation for cooperating with others elicited a higher rate of trusting choices from subjects.

Because Social Exchange theory and Group Norm Maintenance theory both predict this result, it alone does not discriminate the rival theories. It does, however, demonstrate that subjects successfully registered the reputational information, that they changed their behavior toward the partner on the basis of it, and that they did so in a quantitative way (the more often their partner disclosed willingness to mistreat 3^rd^ parties, the less they were trusted by the subject). This means that the results below cannot be explained by arguing that the prior negative behavior of partners was not registered or that subjects did not or could not behave differentially on the basis of it.

### When 1^st^ and 3^rd^ Party Reputation are Both Available, does 3^rd^ Party Reputation Play any Role in Regulating Decisions to Trust?

No. In Study 2, the experimenter simultaneously provided the subject with information about how the partner behaved toward the subject (1^st^ party reputation) and toward another person (3^rd^ party reputation) in earlier PDs. When this was true, 3^rd^ party reputation did not regulate trust at all. In the Study 2 game, subjects mistrusted their partner 12% of the time. Decisions to trust or mistrust were regulated only by 1^st^ party reputation (OR = 2.77, *t_470_* = 3.79, *p* = 10^−4^); third party reputation had no effect (OR = 1.04, *t_470_* = 0.18, *p* = .86).

This result is uniquely predicted by Social Exchange theory. According to Social Exchange theory, reputational information is used to estimate how likely a partner is to cooperate with oneself. First party reputation should override 3^rd^ party reputation because 1^st^ party reputation is a more reliable cue to how the partner will treat the *subject*. Third party reputation is an inferior cue, which depends on the inference that the partner’s cooperative motivations do not discriminate between oneself and others; generally, it should be used only when 1^st^ party information is unavailable or unreliable.

This result is difficult to reconcile with Group Norm Maintenance. It predicts that 1^st^ and 3^rd^ party reputation should jointly regulate decisions to trust, because they both reveal information about the partner’s disposition to violate norms. The target of defection should not matter. Yet the data show that 3^rd^ party reputation plays no role when 1^st^ party information is available. This is not because 3^rd^ party reputation is always ignored. As Study 1 demonstrated, subjects can and do use 3^rd^ party reputation in regulating the decision to trust–they just ignore this larger observational sample when 1^st^ party information is available.

The more powerful effect of 1^st^ party reputation cannot be explained by claiming that subjects have more direct and certain information about norm violations against themselves than against 3^rd^ parties. In Study 2, the reliability of evidence about 1^st^ and 3^rd^ party reputation, including its source and format, was held constant. Subjects learned whether their partner had previously defected in a PD with the subject (1^st^ party) and with another person (3^rd^ party) simultaneously, on the same screen, from the same source, and in the same format. Despite the complete isomorphism of 1^st^ and 3^rd^ party reputational information in Study 2, the mind treated them differently.

### Does 3^rd^ Party Reputation Modulate Subjects’ Willingness to Punish Partners Who Defected on them in the Trust Game?

No. Subjects punished 47% of defecting partners in Study 1, and 64% of defecting partners in Study 2. But, the probability of punishing partners who defected on the subject in the trust game was not affected by 3^rd^ party reputation in either study (Study 1: OR = 0.98, *t_107_* =  −0.27, *p* = .79; Study 2: OR = 1.03, *t_202_* = 0.14, *p* = .89). (It was not affected by 1^st^ party reputation either (Study 2: OR = 0.77, *t_202_* =  −1.14, *p* = .26).) If motivations to punish were shaped by selection pressures to maintain group norms, then reputation for violating group norms should have had an effect. It did not.

We know from Study 1 that subjects can and do use 3^rd^ party reputation to distinguish partners and moderate their behavior accordingly: the more often partners expressed a willingness to cheat 3^rd^ parties, the less they were trusted by subjects. Nevertheless, this same information–explicit information about their partner’s inclination to violate norms–did not regulate the probability of subjects punishing partners who defected on them. The fact that treatment of 3^rd^ parties failed to regulate punishment in either study is difficult to reconcile with the core Group Norm Maintenance claim that punishment is directed at norm violators.

If punishment is not for targeting norm violation, what predicts when it will be deployed? When a partner defects in the first round, but trusts in the second, Social Exchange and Group Norm Maintenance theories make different predictions about how the mechanisms will respond. The best response under Social Exchange depends upon whether the subject chooses to continue the relationship but bargain for better terms or, instead, chooses to cut his or her losses by discontinuing the relationship. If the choice is to continue, the subject is predicted to: (i) punish the defector in the first round (communicating that the partner’s current treatment is unacceptable and incentivizing an improvement), and then (ii) cooperate when the partner trusts in the second round (thereby continuing the relationship). Alternatively, if the choice is to discontinue the relationship, the subject is predicted to: (i) refrain from punishing the defector in the first round (as this leads to a higher payoff) and then (ii) defect in the second round despite the partner’s trust. That is, Social Exchange predicts a strong *positive* association between punishment of a defector and subsequent cooperation with that defector. In contrast, Group Norm Maintenance predicts that if a defector is categorized as a group-norm violator and thus targeted for punishment, that violator should also be targeted for reduced acceptance of cooperative solicitations [43]. Group Norm Maintenance, therefore, predicts a *negative* association between punishing a defector and subsequently cooperating with that defector.

### Does Punishing a Defector Predict a Disposition to Later Cooperate with them?

Yes. Subjects cooperated with 81% of partners in Study 1 and 83% of partners in Study 2. After being defected on in the first round with a particular partner, subjects were far more likely to respond to a later act of trust with cooperation if they had punished the initial defection than if they had not. This was true in both studies (Study 1: OR = 12.59, *t_218_* = 6.20, *p* = 10^−8^; Study 2: OR = 11.04, *t_413_* = 6.40, *p = *10^−9^). Indeed, subjects were *just as likely* to cooperate in round two with a defector whom they had punished in round 1 as with a partner who had cooperated in round 1 (Study 1: OR = 1.13, *t_218_* = 0.24, *p* = .81; Study 2: OR = 0.72, *t_413_* =  −0.92, *p = *.36). This pattern does not make sense if punishment is designed to reduce rates of norm-violation within the group; it makes sense only if punishment is bargaining, targeted at those with whom one plans to attempt to cooperate in the future.

These results are predicted by the social exchange theory that punishment in small-scale cooperative contexts is a form of bargaining in the service of maintaining interpersonal cooperative relationships. Moreover, the increased disposition to cooperate with defectors one has singled out for punishment, while ignoring how they treated third parties, is highly inconsistent with Group Norm Maintenance theories. In many Group Norm Maintenance models, cooperation is *never* extended to individuals who have been categorized as norm violators, because refusal to cooperate is merely a less expensive form of punishment [43]. Indeed, if both behaviors were designed to discourage group norm violations, then subjects who punished their partner's defection because they interpreted it as a norm violation should be *less* willing to reward that defector/norm-violator by cooperating with them in round two. Yet the results show the opposite relationship: Punishment of a round one defector was associated with *willingness* to cooperate with that defector in round two.

### Is the Decision to Cooperate with a Partner Influenced by the Partner’s Past Reputation when his or her most Recent Behavior is Available as a Cue?

No. Because partners always trusted the subject in round 2, we can see whether the subject’s response–cooperation versus defection–is regulated more strongly by the partner’s past reputation or by how the partner treated the subject in round 1. In round 2, subjects were more likely to cooperate with partners who cooperated with them in round 1 of the trust game (Study 1: OR = 11.12, *t_218_* = 4.93, *p* = 10^−5^; Study 2: OR = 8.00, *t_413_* = 6.56, *p* = 10^−9^). Importantly, given a partner’s round 1 behavior, subjects do not choose to cooperate more with partners with positive past reputations, either 3^rd^ party (Study 1: OR = 1.04, *t_218_* = 0.40, *p* = .69; Study 2: OR = 0.57, *t_413_* =  −2.77, *p* = .01, counter-predicted direction) or 1^st^ party (OR = 1.09, *t_413_* = 0.39, *p* = .70).

The fact that past reputation is completely ignored when subjects have a superior cue–the partner’s most recent behavior toward the subject– supports a distinctive prediction of Social Exchange theory (Social Exchange 3): the decision system is sifting for individuals who will profitably cooperate with the actor, not for group norm upholders. Consequently, it switches to the best available cue of partner profitability to make decisions about whether to cooperate.

In contrast, if the function of reputation (as well as the extension of trust) is to defend group norms, then this predicts that information about treatment of 3^rd^ parties should continue to play a role in cooperative decision-making–a role at least equal to a single act of cooperation or defection toward the subject (Group Norm Maintenance 3). This prediction was disconfirmed.

### Conclusions

The debate about whether key cooperative phenomena in humans are the result of a psychology of social exchange or group norm maintenance has persisted because many studies have used methods in which the two theories make overlapping predictions (and so the results can be interpreted as supporting either theory). In contrast, the experiments reported here were designed so that the two theories would make distinct and often opposing predictions. We employed standard cooperative games and two very different forms of reputation, both to replicate the core findings and to ensure that results were robust to differences in the reputational stimuli–that there was nothing about the specific stimuli that gave rise to spurious results.

Subjects were given extensive evidence about their partners’ histories of norm violation, and they had ample opportunities to act on this information. Despite this, they did not deliver more punishment to partners who were more norm-violating–the central prediction of Group Norm Maintenance theory. Instead, they appear to self-interestedly use punishment to bargain for better treatment in individual exchange relationships, by differentially targeting for reform defecting partners with whom they later attempt to continue to cooperate. They inhibit expending punitive effort to reform defectors with whom they themselves will not be cooperating–even though an unreformed norm violator may go on to exploit others.

Refusal to trust a norm violator could be construed as an alternative way of upholding norms. But in deciding whether to trust–and in deciding which acts of trust to reward with cooperation– subjects ignored their partners’ history of mistreating others (norm violation) when they had access to information about how the partner treated them specifically. 1^st^ party reputation trumped 3^rd^ party reputation, and recent behavior toward the self trumped past reputation for norm violation. This is a decision rule that in real social ecologies would lead to higher average individual payoffs by allowing the individual to avoid trusting those who are likely to defect on them.

What Group Norm Maintenance theories survive these results? No finite set of experiments can rule out all alternative hypotheses; the studies presented here were designed to critically test core elements of theory-space. Although existing Group Norm Maintenance theories fare poorly, it is conceivable that new Group Norm Maintenance theories could be constructed which conform to present results. They would, however, have to incorporate rather implausible properties that are very different from theories advanced to date. For example, a Group Norm Maintenance theory might be able to fit this pattern of results if it predicted that humans have only a one-back memory for norm violation, that norm violations against the self render norm violations against others irrelevant (rather than compounding them), and that punishment cleanses norm violator status in ways that spontaneous trusting behavior does not. If such major modifications were introduced to Group Norm Maintenance theory in order to retrodict these findings, they would all but remove the “group” from group norm maintenance theory.

Taken together, these results suggest that interactions between pairs of individuals are strongly shaped by a social exchange psychology that evolved to directly benefit the interactants over the long run. A strong bias to trust; the use of cooperative reputation to initially decide which partners to trust; placing greater weight on how the partner treated you versus others in making decisions to trust, cooperate, and punish; the replacement of reputational cues by direct experience to regulate subsequent interactions; the use of punishment as a bargaining tool when you plan to continue the relationship–these features all fit together as an efficient architecture for small scale social exchange, rather than large scale norm maintenance. It is possible to argue that both psychologies coexist. However, these experiments show that under conditions strongly favorable to the elicitation of group norm maintenance phenomena (exposure to individuals who vary in their norm violation, past and present) these phenomena failed to materialize. This is not a claim that small-scale social exchange exhausts the cooperative selection pressures that have shaped human psychology. Other work, for example, clearly demonstrates cognitive and motivational adaptations for reasoning about coalitions [44], managing coalition membership [45], and engaging in n-party exchange [46]. Rather, the current research complements this existing literature by showing that the psychologies of reputation and punishment are regulated in ways that indicate functional design for social exchange, rather than for group benefits.

## Supporting Information

Appendix S1 **Reputation Instruments.**
(DOCX)

Appendix S2 **Credulity Measures.**
(DOCX)

Appendix S3 **Data Coding Scheme and Results.**
(DOCX)
