# Supplementary material for: What Are Punishment and Reputation for?
Source: PLoS One. 2012 Sep 26;7(9):e45662. doi: 10.1371/journal.pone.0045662 (PMC3458883; doi:10.1371/journal.pone.0045662)
Supplement: Appendix S1 — Reputation Instruments. (DOCX) [file pone.0045662.s001.docx]

**Appendix S1: Reputation Instruments**

*Character Assessment: Study 1*

Subjects were asked to read the following scenarios and answer honestly how they would behave in each.

Cooperative Disposition Questionnaire

1) You need to get gas before going to pick up your friends to go out to dinner. When you get to the pump you see a sign that says “If paying by credit card, slide your card after pumping your gas.” A lot of people are getting gas while you are there. As you finish pumping your gas, the power goes out and all the pumps stop working. You look around and see that some of the other people are going inside to pay since the pay-at-the-pump option is no longer working. What would you do?

Option 1: I would go inside to pay

Option 2: I would leave without paying

2) You are renting a house with three other people. During your first week living together, you and your housemates made a list of twelve chores that need to be done every week. Everyone is supposed to do three of the chores. Each week, a new copy of the chore list is posted in the kitchen, and each person crosses out a chore after he or she completes it. To motivate yourselves, you and your housemates agreed that when someone doesn't do their three chores, that person will have to do double the next week. At the end of last week, “Take out the trash” remained on the list. Someone had not completed three chores. You think back and realize that you had only done two chores during the week. You don't want to have to do double next week. Your housemate asks everyone, “Okay, who didn't do three?” What would you do?

Option 1: I would insist that I had done my three chores

Option 2: I would admit that I had done only two chores

3) Whenever you go back to your home town, you have lunch at a nice restaurant with your friend Jesse from high school. You and Jesse have a tradition of alternating on who picks up the tab. The last time you were home, Jesse paid for lunch. You are home again, and have just finished a nice, leisurely, expensive lunch with Jesse. Jesse reaches for the check, saying, “Hey, this time it's my turn; you paid last time!” Obviously, Jesse has forgotten that it is your turn. What would you do?

Option 1: I would say nothing, and let Jesse pay the bill

Option 2: I would tell Jesse that it is my turn this time, and pay the bill

4) Six months ago, you landed a job as a reporter for the local newspaper. One of your older co-workers, Sarah, has been great - she's been teaching you a lot and explaining what your very demanding editor expects of your articles. On many nights she has stayed late, giving you advice and helping you rewrite the leads to your articles before your editor sees them. Tonight, Sarah comes to your desk, looking frantic. She tells you that she has an urgent situation to take care of at home, and she desperately needs you to finish her article for her. The problem is, tonight is your first date with someone you have been attracted to for a long time. What would you do?

Option 1: I would cancel my date and help Sarah

Option 2: I would go on my date and not help Sarah

5) You are playing in an intramural tennis tournament. In this tournament there are no official referees, and the players make their own calls. You made it to the semifinals and know you have a good chance of winning the whole thing. In the semifinal match, you and your opponent are both strong players, and the match is exhausting. You finally work your way to match point; one more point and you will win. But if your opponent wins the point, you will probably be playing for another half an hour. The ball is served and you hit a good return. Your opponent mis-hits the ball and it sails toward the far corner of the court. You race after it, just happening to block the ball from your opponent's view, and watch as it just hits the line. It was in, but you were a step away from being able to take a good swing at it. What would you do?

Option 1: I would lie and tell my opponent that the ball was out

Option 2: I would admit to my opponent that the ball was in

6) You are approaching a deadline on an advertising campaign. But you have to leave the office before finishing the job. Your co-worker offered to fill in for you and finish working on the campaign. Your co-worker then spent half the night finishing the job. The next morning, you arrive at work before your co-worker does. Your superiors are impressed; they compliment you on your hard work on the advertising campaign. What would you do?

Option 1: I would tell them that it was actually my co-worker who had stayed late to finish the campaign.

Option 2: I would thank them for the compliment, saying how tired I was from having stayed so late to finish it

Prior to each trust game subjects were provided with the complete text of four questions along with the answers chosen by their partner.

*Character Assessment: Study 2*

Subjects were matched with four partners and played a Prisoner’s Dilemma (PD) game with each. In each game, subjects were endowed with 25 points. They could choose between keeping the points or giving them to their partner, in which case the points given to the partner would triple to 75. Both partners would earn the most points jointly if both gave their initial endowment. However, each subject could earn the most points individually if they kept their endowment and their partner gave them their endowment. Subjects did not receive feedback regarding their partner's decisions until the Trust Game portion of the experiment. (Payoffs expressed as: Subject, Partner)

|  |  | **Partner Decision** | |
| --- | --- | --- | --- |
|  |  | **Give** | **Keep** |
| **Subject**  **Decision** | **Give** | 75, 75 | 0, 100 |
|  | **Keep** | 100, 0 | 25, 25 |

For each trust game, subjects were provided with two of their partner’s PD game decisions.

Example:

Your partner: Player 7

Player 7: GAVE the points when partnered with You

Player 7: KEPT the points when partnered with Player 3
