# Supplementary material for: What Are Punishment and Reputation for?
Source: PLoS One. 2012 Sep 26;7(9):e45662. doi: 10.1371/journal.pone.0045662 (PMC3458883; doi:10.1371/journal.pone.0045662)
Supplement: Appendix S2 — Credulity Measures. (DOCX) [file pone.0045662.s002.docx]

**Appendix S2: Credulity Measures**

*Credulity Check: Study 1*

Following the experiment, subjects were verbally probed for suspicion of the sham partner deception (loosely asking, "Was there anything you found odd about the study? Did you think that anything was not as it was described?"). Any mention of suspicion of the deception resulted in the subject being coded as suspicious. Thirty eight subjects (25 females) met this criterion.

*Credulity Check: Study 2*

Following the experiment, subjects were probed for suspicion of deception with a six question debriefing. Without explicitly mentioning the nature of the deception—to avoid the demand characteristics of such a question—the debriefing was structured to implicitly probe for suspicion of the deception, as well as when the suspicion occurred and what effects the subject believed it may have had. These questions asked:

1) What questions do you have about the study to this point?

2) Was their anything unclear about the study? If so, What?

3) Has anything struck you as odd about the study? If so, what?

4) Do you think there was more to this study than was described in the instructions? If so, what?

5) When did these thoughts occur to you?

6) Do you think these thoughts influenced your decisions? If so, how?

If at any point during the debriefing subjects mentioned any suspicion that they were not partnered with real humans, or that the information they were given was fabricated in any way, they were coded as suspicious of the deception. Twenty four subjects (14 females) met this criterion.
