# Supplementary material for: What Are Punishment and Reputation for?
Source: PLoS One. 2012 Sep 26;7(9):e45662. doi: 10.1371/journal.pone.0045662 (PMC3458883; doi:10.1371/journal.pone.0045662)
Supplement: Appendix S3 — Data Coding Scheme and Results. (DOCX) [file pone.0045662.s003.docx]

**Appendix S3: Data Coding Scheme and Results**

*Data Analysis: Coding schemes*

Sex is coded: 1 for females, 0 for males

Credulity is coded: 1 for credulous subjects, 0 for suspicious subjects

Character assessment is coded as:

Study 1: The number of questions for which the subject picked the cooperative option on the cooperative disposition questionnaire (0-6).

Study 2: The number of PD games for which the subject gave their endowment to their partner (0-4).

3rd Party Reputation is coded as:

Study 1: The number of times the partner reported they would cooperate with 3rd parties in the social dilemma questionnaire (0, 1, 3 or 4).

Study 2: The number of times the partner was reported to have transferred their endowment to partners besides the subject in the previous PDs (0, 1 or 2).

1st Party Reputation is coded as:

Study 2: The number of times the partner was reported to have transferred their endowment to the subject in the previous PDs (0 or 1).

Trust/Distrust is coded: 1 for trust, 0 for distrust

^[[1]](#footnote-1)^Cooperation is coded: 1 if the partner cooperated, and 0 if the sham partner defected.

^1^Punished Defection is coded: 1 if the partner defected and was punished, and 0 otherwise.

^1^Unpunished Defection is coded: 1 if the partner defected and was not punished, and 0 otherwise.

Cooperate/Defect is coded: 1 if the subject cooperated in round 2, 0 if they defected.

**Study 1 Results**
(population-averaged, non-linear models with logit link function and robust standard errors)

*Table 1.* HLM of Decisions to Trust / Distrust (Round 1)

Effect b S.E. Odds Ratio t df p

Between-Subjects Effects

Intercept 0.545 0.154 1.725 3.532 89 .001

Sex -0.482 0.323 0.617 -1.491 89 .139

Credulity -0.173 0.312 0.841 -0.553 89 .581

Character Assessment 0.112 0.165 1.119 0.678 89 .499

Within-Subjects Effects

3rd Party Reputation 0.486 0.069 1.626 7.010 367 ***

*Table 2.* HLM of Decisions to Punish / Not (Round 1)

Effect b S.E. Odds Ratio t df p

Between-Subjects Effects

Intercept -0.125 0.226 0.883 -0.551 70 .583

Sex -0.662 0.473 0.752 -1.399 70 .166

Credulity -0.285 0.438 0.752 -0.650 70 .518

Character Assessment 0.134 0.215 1.143 0.622 70 .536

Within-Subjects Effects

3rd Party Reputation 0.014 0.097 1.014 0.145 109 .885

*Table 3.* HLM of Decisions to Cooperate / Defect (Round 2)

Effect b S.E. Odds Ratio t df p

Between-Subjects Effects

Intercept 1.816 0.210 6.146 8.635 81 ***

Sex -0.141 0.444 0.868 -0.318 81 .751

Credulity 0.371 0.414 1.449 0.897 81 .373

Character Assessment 0.179 0.167 1.195 1.068 81 .289

Within-Subjects Effects

3rd Party Reputation 0.021 0.090 1.021 0.234 222 .816

Cooperation vs. 2.517 0.407 12.394 6.182 222 ***

Unpunished Defection

Punished Defection vs. 2.391 0.481 10.919 4.973 222 ***

Unpunished Defection

Punished Defection vs. -0.127 0.511 0.881 -0.248 222 .804

Cooperation

Study 2 Results
(population-averaged, non-linear models with logit link function and robust standard errors)

*Table 4.* HLM of Decisions to Trust / Distrust (Round 1)

Effect b S.E. Odds Ratio t df p

Between-Subjects Effects

Intercept 2.288 0.177 9.858 12.869 115 ***

Sex -1.395 0.373 0.248 -3.742 115 ***

Credulity 0.307 0.339 1.360 0.908 115 .366

Character Assessment 0.089 0.115 1.093 0.775 115 .440

Within-Subjects Effects

1st Party Reputation 1.020 0.269 2.772 3.793 470 ***

3rd Party Reputation 0.043 0.243 1.044 0.178 470 .859

*Table 5.* HLM of Decisions to Punish / Not (Round 1)

Effect b S.E. Odds Ratio t df p

Between-Subjects Effects

Intercept 0.614 0.184 1.848 3.341 101 .002

Sex 0.042 0.376 1.043 0.111 101 .912

Credulity -0.232 0.468 0.793 -0.495 101 .621

Character Assessment -0.121 0.131 0.886 -0.929 101 .355

Within-Subjects Effects

1st Party Reputation -0.266 0.235 0.766 -1.135 202 .258

3rd Party Reputation 0.032 0.224 1.032 0.142 202 .888

*Table 6.* HLM of Decisions to Cooperate / Defect (Round 2)

Effect b S.E. Odds Ratio t df p

Between-Subjects Effects

Intercept 1.942 0.167 6.976 11.617 115 ***

Sex 0.319 0.318 1.376 1.004 115 .318

Credulity -0.540 0.487 0.583 -1.110 115 .270

Character Assessment 0.562 0.112 1.755 4.999 115 ***

Within-Subjects Effects

1st Party Reputation 0.084 0.217 1.088 0.388 413 .698

3rd Party Reputation -0.567 0.205 0.567 -2.765 413 .006

Cooperation vs. 2.080 0.317 8.001 6.564 413 ***

Unpunished Defection

Punished Defection vs. 2.402 0.375 11.040 6.400 413 ***

Unpunished Defection

Punished Defection vs. 0.322 0.350 1.380 0.919 413 .359

Cooperation

Study 1 Results (Credulous Subjects Only)
(population-averaged, non-linear models with logit link function and robust standard errors)

*Table 7.* HLM of Decisions to Trust / Distrust (Round 1)

Effect b S.E. Odds Ratio t df p

Between-Subjects Effects

Intercept 0.465 0.199 1.591 2.329 52 .024

Sex -0.344 0.439 0.709 -0.784 52 .437

Character Assessment 0.033 0.209 1.033 0.156 52 .877

Within-Subjects Effects

3rd Party Reputation 0.479 0.088 1.614 5.418 216 ***

*Table 8.* HLM of Decisions to Punish / Not (Round 1)

Effect b S.E. Odds Ratio t df p

Between-Subjects Effects

Intercept -0.225 0.306 0.798 -0.735 40 .466

Sex -0.498 0.635 0.608 -0.784 40 .438

Character Assessment 0.097 0.243 1.102 0.401 40 .690

Within-Subjects Effects

3rd Party Reputation 0.005 0.150 1.005 0.031 61 .976

*Table 9.* HLM of Decisions to Cooperate / Defect (Round 2)

Effect b S.E. Odds Ratio t df p

Between-Subjects Effects

Intercept 2.106 0.300 8.217 7.014 47 ***

Sex 0.147 0.579 1.159 0.254 47 .801

Character Assessment 0.174 0.225 1.190 0.774 47 .443

Within-Subjects Effects

3rd Party Reputation 0.169 0.148 1.184 1.137 126 .258

Cooperation vs. 3.059 0.544 21.302 5.621 126 ***

Unpunished Defection

Punished Defection vs. 3.273 0.835 26.397 3.919 126 ***

Unpunished Defection

Punished Defection vs. 0.214 0.869 1.239 0.247 126 .805

Cooperation

Study 2 Results (Credulous Subjects Only)
(population-averaged, non-linear models with logit link function and robust standard errors)

*Table 10.* HLM of Decisions to Trust / Distrust (Round 1)

Effect b S.E. Odds Ratio t df p

Between-Subjects Effects

Intercept 2.385 0.219 10.863 10.892 92 ***

Sex -1.506 0.468 0.222 -3.216 92 .002

Character Assessment -0.023 0.139 0.977 -0.164 92 .871

Within-Subjects Effects

1st Party Reputation 1.172 0.297 3.227 3.947 375 ***

3rd Party Reputation 0.000 0.265 1.000 0.000 375 1.000

*Table 11.* HLM of Decisions to Punish / Not (Round 1)

Effect b S.E. Odds Ratio t df p

Between-Subjects Effects

Intercept 0.574 0.204 1.776 2.817 81 .007

Sex -0.092 0.417 0.912 -0.220 81 .826

Character Assessment -0.075 0.144 0.928 -0.519 81 .605

Within-Subjects Effects

1st Party Reputation -0.236 0.268 0.790 -0.881 165 .380

3rd Party Reputation -0.013 0.252 0.988 -0.050 165 .961

*Table 12.* HLM of Decisions to Cooperate / Defect (Round 2)

Effect b S.E. Odds Ratio t df p

Between-Subjects Effects

Intercept 1.899 0.182 6.680 10.459 92 ***

Sex 0.859 0.354 2.361 2.428 92 .017

Character Assessment 0.643 0.125 1.901 5.128 92 ***

Within-Subjects Effects

1st Party Reputation 0.063 0.254 1.065 0.249 331 .804

3rd Party Reputation -0.408 0.227 0.665 -1.801 331 .072

Cooperation vs. 2.324 0.349 10.220 6.656 331 ***

Unpunished Defection

Punished Defection vs. 3.082 0.460 21.807 6.669 331 ***

Unpunished Defection

Punished Defection vs. 0.758 0.410 2.134 1.847 331 .065

Cooperation

1. Together, "Cooperation" and "Punished Defection" form a contrast set which compares the differences between these two outcomes (cooperation and punished defection) to the contrast category of unpunished defection. 'Unpunished Defection' then replaces 'Cooperation' to calculate the final contrast between cases where the sham partner cooperated and when they defected but were punished. [↑](#footnote-ref-1)
